# Rethinking the Role of Affect in Risk Judgment: What We Have Learned From COVID-19 During the First Week of Quarantine in Italy

**DOI:** 10.3389/fpsyg.2020.554561

**Published:** 2020-10-02

**Authors:** Massimiliano Barattucci, Alice Chirico, Goran Kuvačić, Andrea De Giorgio

**Affiliations:** ^1^Faculty of Psychology, eCampus University, Novedrate, Italy; ^2^Catholic University of the Sacred Heart, Milan, Italy; ^3^Faculty of Kinesiology, University of Split, Split, Croatia

**Keywords:** emotions, anxiety, quarantine, behavior, SARS-CoV-2

## Abstract

Due to COVID-19 spreading in Italy, on March 11 the Prime Minister of Italy declared a lockdown and imposed severe restrictive measures impacting citizens’ freedom at several levels. People were required to stay at home and go out only to satisfy basic needs. Several risk models have postulated a link among online searching behavior, affect, anxiety, and complaints by individuals toward government restrictions (GR), which emerged as also related to an increased perception of knowledge toward risk. However, to date, no study has addressed how these key risk-related aspects (i.e., affect, anxiety, perceived knowledge on risk, and risk dimensions) can act jointly to orient online health information-seeking behavior, and people’s complaints toward GR imposed during the lockdown. This study investigated the mechanisms underlying online health information-seeking behavior and people’s complaints toward the government’s restrictions during a COVID-19 emergency in the Italian population. Drawing from the health belief model (HBM), which postulates a link between sociodemographic variables, risk, and affect dimensions in emergency, we assumed risk factors as predictors of affect and anxiety, which, in turn, were posited as mediators between risk dimensions, online health information-seeking behavior, and complaints toward GR. Participants (1,031) were involved during the first week of the quarantine (March 11–18) and completed an online survey composed of (i) an adapted version of the Italian Risk Perception Questionnaire; (ii) the Italian Positive (PA) and Negative Affect (NA) Schedule (PANAS-10); (iii) the State Anxiety Scale (STAI-Y1); (iv) *ad hoc* personal knowledge measure about novel coronavirus; (v) *ad hoc* item measuring information search behavior regarding the novel coronavirus; (vi) *ad hoc* measure of the complains regarding GR; and (vii) sociodemographic questions. General linear models and structural equation modeling (SEM) were carried out to test the model. Sociodemographic and cognitive factors predicted the participants’ affect and anxiety, which, in turn, motivated and fully mediated both information search behavior and complaint toward GR. This research can offer useful suggestions for policy-makers during the COVID-19 emergency, and it advanced the knowledge on the risk–emotion link in emergency situations.

## General Introduction

In December 2019, a cluster of pneumonia cases of unknown etiology was detected in the city of Wuhan, Hubei Province, central-eastern China. This initial phenomenon turned into a novel coronavirus (Zhu et al., 2020), which is named SARS-CoV-2 (i.e., Severe Acute Respiratory Syndrome), and which caused a disease named COVID-19 (Qu et al., 2020). Even though symptomatology has been defined clearly, it is still hard to define how long it will last and if a cure is possible (Porcheddu et al., 2020; Wangping et al., 2020). Recently, the infection has caused enough deaths to be considered as a pandemic by the World Health Organization (WHO) (Onder et al., 2020; Sohrabi et al., 2020).

In Italy, the outbreak spread on February 20, and after an *ad hoc* decree of the President of the Council of Ministers (DPCM), a lockdown was imposed on Italians (i.e., 20 days after the first recognized patient). All Italians were required to stay home if they were not involved in jobs or tasks involved in other people’s survival. Since March 11 in Italy, restrictive and severe measures have been gradually implemented (from March 11 to 18) (De Giorgio, 2020a,b).

According to the health belief model (HBM) (Janz and Becker, 1984; Carpenter, 2010)—well-established theoretical frameworks in health-related behavior research—often, the psychological counterpart of disease-related emergencies can entail an increased risk perception (Bults et al., 2011) modulated also by sociodemographic variables (e.g., Vaughan, 2011; Clifton et al., 2016). This cognitive perception of risk can have significant implications on individuals’ emotional states on the short and on the long-term (Cafagna and Barattucci, 2019). Moreover, it would be closely related to the intention to adopt protective behaviors (Leppin and Aro, 2009; Goodwin et al., 2011) as well as to personal susceptibility (Lin et al., 2020).

However, HBM has never been used to investigate the mechanisms underlying all these variables in a pandemic situation. Moreover, no data on the Italian population’s risk perception have been reported yet. Crucially, no studies have investigated the impact of risk cognition and emotional response on research behavior and compliance with government actions.

This last aspect can be far more relevant if considering that cognitive perception of risk is sensitive to peculiar emergency-related environmental factors. For instance, Italians were forced to stay home, thus changing their normal habits related to work and leisure activities. Confined at home, Italians tended to rely more on the Internet to remain up-to-date on pandemic progress in a safe way. Crucially, online information searching regarding health issues is not a neutral task since it can influence people’s affective states, especially anxiety (Jutel, 2017).

**FIGURE 1:**
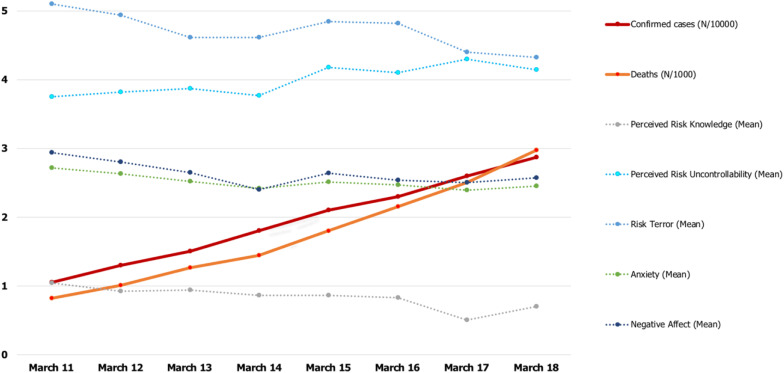
The first week of lockdown in Italy: epidemiological and variable trends. All variables have been standardized. Due to the numerical difference of epidemiological data between Northern and Southern Italy, we divided the real values by 1,000 and have thus reported them in the *y*-axis.

To investigate the joint impact of cognitive risk dimensions, affect, and anxiety on online searching behavior and compliance toward government restrictions (GR), in the peculiar context of the Italian pandemic emergency, we drew from the HBM to formulate and test a novel explicative model. First, we posed the first day of lockdown (March 11, 2020) as the *trigger* event and the online health information on COVID-19 searching behaviors as the main *outcome*. Then, we built and tested a novel model including sociodemographical factors, risk cognitions, behaviors, and affect as mediators between the trigger event and the main outcome of the online health information searching behaviors (Figure 1).

Elucidating this mechanism can be crucial also because information-seeking behaviors can influence the population’ general compliance with government decisions (Clifton et al., 2016). Therefore, these data can provide the government with useful indications regarding which online communication strategies would be the most effective in an emergency situation (Liao et al., 2020).

### Conceptualization of a New Model of Risk Perception

The term “risk” represents the possibility of suffering damage connected to foreseeable circumstances. In essence, it is consequently a variable connected to the frequency (or probability) of the occurrence of the damage and the magnitude that the latter can cause in the individual (Slovic, 2000). This universally recognized definition may look as reducible to a mere mathematical formula. However, its subjective dimension suggests a deeper complexity. Indeed, a plethora of approaches have been developed to capture all the key aspects related to risk perceptions, as well as its main consequences on people’ behavior.

Among the main subjective dimensions of risk, cognitive factors emerged as playing a key role (Slovic, 2000; Leppin and Aro, 2009). Risk perception would be determined by a complex series of cognitive factors: (i) the perceived possibility of having damage to health; (ii) the subjective importance that the damage is more or less possible; (iii) by personal uncertainty associated with the exposure to a specific risk factor (Slovic et al., 2004). In the case of general risk or infection or disease, personal knowledge negatively affects the perception of risk danger (Shook et al., 2019). In turn, risk perception impacts behaviors (Sjöberg, 2000), specifically between different risk dimensions regarding infection, perceived fatality, severity, vulnerability, and uncontrollability, and are proven to have effects on protective conduct (de Zwart et al., 2009). In regard to pandemic-related risk perceptions, two main factors emerged as relevant, that is, vulnerability (a person’s subjective perception of the risk of acquiring an illness or disease) and severity (a person’s feelings on the seriousness of contracting an illness or disease) of harm (Carpenter, 2010). However, despite that it has been repeatedly shown that risk perception can affect behavior (Brewer et al., 2007; Vaughan, 2011; Shook et al., 2019), the underlying mechanism still needs to be elucidated.

Specifically, antecedents of cognitive dimensions of risk should be still clarified. With this regard, demographic factors/variables emerged as playing a key role in shaping pandemic risk perception and subsequent behaviors (see e.g., Vaughan, 2011). For instance, women resulted as more avoidant, fearful, and vulnerable in terms of pandemic risk perception, with lower risk acceptance scores when compared to men (see e.g., de Zwart et al., 2009). Conversely, age often leads to an increased perception of control on infection risk, lower susceptibility, avoidance, and higher acceptance of risk (see e.g., Clifton et al., 2016). Conversely, the level of education was negatively related to the risk of infection and contagion (i.e., vulnerability) (Gidengil et al., 2012). Lower income and urbanization positively affected vulnerability and perceived infection risk (Brewer et al., 2007; De Zwart et al., 2007; Gidengil et al., 2012).

Another factor, which would act as a mediator, should be included between risk perception and behavior, that is, affect. Affect, such as fear, is related to a general amplification of the perception of the danger of risky events, while anger would be significantly associated with underestimation of dangers (Slovic, 2000; Brown, 2014). Moreover, the degree of emotional involvement in the perceived consequences of different risks, or specific personality dimensions that determine emotional attitudes, is associated with different aspects of risk perception (among all, vulnerability, and severity) (Slovic, 2000; Brown, 2014).

Crucially, among the stimuli triggering emotional states, also online searching information should be included, which could also lead to a phenomenon of large-scale emotional contagion (Hatfield et al., 1993). Emotions expressed via the Internet, and mainly through social media, can lead to a long-term psychological impact (Arapakis et al., 2008; Fowler and Christakis, 2008; Coviello et al., 2014; Kramer et al., 2014; Ferrara and Yang, 2015; Mui et al., 2018) including also a simple health information search (Gadahad et al., 2013). Specifically, both general and specific discrete emotional states can orient people’s online search for information on health issues (Wissow, 2007; Myrick and Willoughby, 2019). Emotions and affect act as motivators of specific survival behaviors (Frijda et al., 1989), and this definition could hardly be more appropriate than in this worldwide emergency. In this case, one key survival behavior motivated by affect could consist of online health information seeking or avoidance (Savolainen, 2014). While positive affect (PA) resulted in determining people’s attitudes toward information avoidance, negative affect (NA) predicted individuals’ attitudes toward information seeking (Yang and Kahlor, 2013).

On the other hand, searching for information about symptoms or specific illnesses can increase people’s distress and anxiety about their health (Graffigna et al., 2017). Crucially, NA and anxiety have often resulted in closely positively intertwined affective states (Crawford and Henry, 2004), even though they can be considered as clear, distinguishable constructs (Watson and Kendall, 1989; Clark and Watson, 1991). According to the Tripartite model of anxiety and depression, high levels of NA underlie both anxiety and depression, while NA would act as a central risk factor of anxiety (Clark and Watson, 1991). NA has also often been considered an early predictor of anxiety in several domains (Crawford and Henry, 2004; Cisler et al., 2010). During the lockdown, the Internet became one of the most important sources of health-related information; thus, it would be crucial to analyze antecedents of this behavior as well as its potential impact on compliance with GR.

To date, the literature regarding risk perception and behavior on worldwide pandemics has focused mainly on general population’s or on healthcare workers’ punctual psychological responses immediately after the end of isolation (Wilder-Smith and Freedman, 2020). Acute stress/posttraumatic disorders, as well as higher propensity to live state anxiety, emerged as serious issues (Leppin and Aro, 2009). Crucially, no data on the Italian population’s risk perception have been reported yet. Moreover, no studies have investigated the role of risk cognition and emotional response to research behavior and compliance with government actions.

In this study, we aimed to advance previous studies on COVID-19 at two levels. First, we elucidated the link between cognitive and emotional risk dimensions in a pandemic, then, we built and tested a novel model linking cognitive, emotional, and sociodemographic factors to a peculiar behavior enacted in this emergency, which would be probably increasingly adopted in the future, that is, online searching behavior of health-related information. Moreover, we also used the HBM, for the first time, as a general explicative framework in a pandemic situation.

**FIGURE 2:**
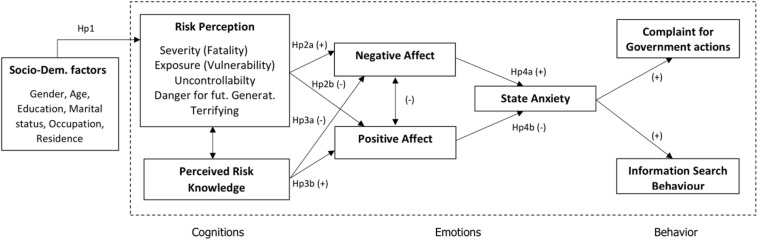
Research model and hypotheses.

Health belief model posits a cognitive appraisal framework, in which perception of the risk for individual health affects emotions and protective behavior (Roseman, 1996). More specifically, when referring to the HBM framework (Janz and Becker, 1984) and adapting recent theoretical models (Watson and Spence, 2007; Keller et al., 2012; Lemée et al., 2019), the present research model considers sociodemographics as antecedents of risk cognition and emotion as a buffering factor between risk perception and behavior (Figure 2).

This novel model proposes that two different risk cognition aspects have independent effects on PA and NA. Perceptions regarding specific pandemic and perceived knowledge of risk (Champion and Skinner, 2008; Carpenter, 2010) can act differently on contingent affect, which can have an impact on both information search behavior and complaints regarding government action. In a situation of physical and social constraint, i.e., quarantine, the sudden perceptions of the risk would depend mainly on mass media, social media, and word of mouth information (Jung et al., 2015). This growing information impacting the emotional state can, in turn, act as both a search trigger for further infection information and a facilitator of compliance with the government’s restrictions (Goodwin et al., 2011; Rolison and Hanoch, 2015).

In line with HBM and literature, major evidence linking cognitive risk dimensions and affect (de Zwart et al., 2009; Keller et al., 2012), this study aimed to explore the following hypotheses: sociodemographic factors have an impact on risk perception and perceived risk knowledge (Hp1); more precisely, the research expects that age (Hp1a) and education (Hp1b) will negatively affect risk perception and positively risk knowledge; thus, it is hypothesized that women will have a worse perception of pandemics and less perceived knowledge compared to men (Hp1c). The research assumed that risk perception would positively impact on NA (Hp2a) and negatively on PA (Hp2b); on the contrary, it expected that risk knowledge would negatively impact on NA (Hp3a) and positively on PA (Hp3b). Moreover, the research intends to elucidate whether the differential effect of NA (Hp4a) and PA (Hp4b) on search behavior and a complaint is mediated by state anxiety (Hp4). In order to test all the mentioned hypotheses thoroughly, we tested this novel model by means of structural equation modeling (SEM) (Figure 2).

## Materials and Methods

### Participants

One thousand thirty-one participants from Italy voluntarily took part to this study (mean age = 38.34; SD = 13.02, range = 18–82). After removing the data of the participants who did not answer all the survey questions, we analyzed 998 participants, of which 739 were females (mean age = 37.01; SD = 12.39) and 259 were males (mean age = 42.15; SD = 14). Their marital status was as follows: 37.68% were engaged in a relationship, 32.57% were married, 23.75% were single, 5.42% were divorced, 0.6% were widowed; 23.75% reported living in central big city areas, 20.4% were living in the suburb of a big city, 36.07% reported living in a small town (i.e., less than 50,000 inhabitants), and 20.14% reported living in the countryside; 57.52% resided in Northern Italy, 17.33% resided in central Italy, 17.64% resided in South Italy, and 7.52% resided in the Islands; 20.44% were students, 2.51% were retired, 25.35% were freelance, 11.72% were temporary workers, and 30.96% were full-time employees with a permanent position; and 52.93% reported having no children, 17.13% reported having two children, 16.43% reported having one child, and 0.6% reported having more than three children.

Regarding schooling, 3.71% reported having a middle school diploma, 31.16% declared having a high school degree, 18.84% reported having a bachelor’s degree, 24.15% reported having a master’s degree, and 21.64% reported having a Ph.D.

This study was conducted in accordance with APA ethical standards and with the Declaration of Helsinki. Participants: (i) were fully informed in regard to institutional affiliations of the researchers and research scope; (ii) continued the survey only if they were adult (>18 years old); (iii) gave information that could not allow their identification; (iv) had the right to refuse to participate in the study and withdraw at any time; (v) filled an anonymous questionnaire and confirmed the understanding of instructions and voluntary participation.

### Procedures and Materials

Participants completed an online survey between March 11, the first day of quarantine and national lockdown, and March 18. The research design relied on snowball sampling (chain referral process). Participants were recruited through flyers, social networks, and by word of mouth. The questionnaire answering began in the evening (March 11) when the DPCM decree was issued. First, participants completed the part of the questionnaire created to gather sociodemographic information. Second, the following questionnaires were then completed:

1. *Italian Risk Perception Questionnaire* (Cafagna and Barattucci, 2019): originally developed by Savadori et al. (1998). Based on literature indications (Keller et al., 2012), the study deduced that the pandemic risk could generally be identified as terrifying, uncontrollable, fatal, and dangerous for future generations, and widespread in terms of exposure. Hereupon, the researchers built a tool that measures five single-item dimensions of risk infection, on a seven-point scale ranging from 1 to 7: severity, vulnerability, uncontrollability, terror, and danger for future generation (item examples: “Considering the scale below (1 ‘not fatal’ to 7 ‘fatal’): in your opinion, when the virus infects a subject, how likely are the consequences of being fatal?” “Considering the scale below (1 ‘not exposed’ to 7 ‘totally exposed’), in your opinion, to what extent do you think you are exposed to the coronavirus risk?”
2. *Italian short version of the Positive and Negative Affect Schedule* (Terraciano et al., 2003): a 10-item self-report scale on a five-point Likert scale, which captures the two main clusters of the current affective experience, i.e., positive (five adjectives; Cronbach’s alpha = 0.790) and negative affect (five adjectives; Cronbach’s alpha = 0.940).
3. *State Anxiety Scale (STAI-Y1)*: a 20-item self-report questionnaire on a four-point Likert scale (from 1 “not at all” to 4 “very much so”; original Cronbach’s alpha = 0.954) to assess participants’ current state of anxiety.
4. *Ad hoc* measure about *Novel coronavirus personal knowledge*, which tests how people know the disease: “Do you know exactly the difference between COVID-19 and SARS-CoV-2?” This measurement tool consists of a three-level ordinal scale: (i) Yes, I know it perfectly. (ii) Yes, I know generally. (iii) No.
5. *Ad hoc Information search behavior* item regarding novel coronavirus: “Thinking about the last week, how many times did you search on the Internet (Google, news, articles on social networks, etc.) for information on COVID-19, SARS-CoV-2, or Coronavirus?” This measurement tool consists of a four-level ordinal scale: (i) never, (ii) sometimes, (iii) several times, (iv) often, and (v) everyday.
6. *Ad hoc Complaint regarding government actions* item: “The Government has acted late to contain the spread of the virus.” This measurement tool consists of a four-level ordinal scale of accordance with the phrase: (i) I totally disagree. (ii) I disagree a little. (iii) I mostly agree. (iv) I totally agree.

### Data Analysis

A Kolmogorov–Smirnov test for each of the variables involved in this study was carried out to test their distribution. We found that all target variables (i.e., anxiety, NA, risk dimensions, online health information searching behavior, and the complaint about government measures) were normally distributed. To test the causal relationship between all demographical variables (i.e., marital status, job position, age, gender, residence area, and residence area in Italy) and each target variable, a generalized linear model (GLM) for categorical and ordinal data was carried out with SPSS Ver. 21.0 (IBM Co., Armonk, NY, United States) statistical program. The GML is robust to the violation of sphericity as it does not necessarily assume a normal distribution of variables (Agresti and Kateri, 2011). Moreover, regarding the residence area, we operationalized the “residence area” into two different variables. The former “residence area” refers to how far from the city center a person lives: (i) city center; (ii) suburb of a city; (iii) town; (vi) countryside, and (v) the latter, “residence area in Italy” refers to a zone of residence from the north to the south of Italy and islands, which also coincides with the distance from the first epidemic center of diffusion (i.e., Codogno) in Northern Italy. A comprehensive structural equation model with AMOS22 was used to test the proposed theoretical model and the main hypotheses. Commonly reported fit statistics were: comparative fit index (CFI), Tucker–Lewis index (TLI), normed fit index (NFI), goodness of fit index (GFI), incremental fit index (IFI), root mean square error of approximation (RMSEA), plus standardized root mean square residual (SRM) for measurement model fit. Research has sought to reduce response bias and common-method variance problems utilizing suggested methods (Podsakoff et al., 2012): scales were visually divided, and different formats and endpoints were used for each different measure.

## Results

### Sociodemographic Variables and Psychological Impact on Anxiety, Negative Affect, Risk Dimensions, and Search Behavior

All factors are reported in Table 1 with significant regression coefficients and Wald statistics. All Omnibus models were significant except for the model with “Knowledge,” i.e., a risk dimension, as the predicted variable.

**TABLE 1:** Generalized linear model multiple regressions with gender, marital status, education, number of children, residency, residency in Italy and age as predictors and anxiety, positive affect, negative affect, search behavior, knowledge of COVID-19, vulnerability, control, severity, risk as terrifying, risk as damage for future generations, and complaint to Government’s measures as predicted variables.

**Predicted variables**											

**Predictors**	**Statistics**	**Anxiety**	**PA**	**NA**	**SB**	**KNW**	**VUL^*a*^**	**CON**	**SEV**	**TER**	**DFG**	**COM**
Gender: female	B	4.156	−1.367	1.373	–	12.75	–	–	0.625	–	0.567	0.186
	Wald χ^2^	19.479	0.097	11.09	–	0.389	–	–	49.03	–	21.858	9.51
	Significant	*p* < 0.001	*p* < 0.001	*p* = 0.001	–	*p* < 0.001	–	–	*p* < 0.001	–	*p* > 0.001	0.002
	CI_95_	2.31; 6.01	−0.442;.609	0.557; 2.189	–	0.175; 0.602	–	–	−0.45; 0.80	–	0.372; 0.85	0.068; 0.304
Gender: male	Redundant											
Status: engaged (married, in a relationship)	B	–	–	−0.963	−0.26	–	−0.21	–	–	–	–	–
	Wald χ^2^	–	–	5.82	7.610	–	3.821	–	–	–	–	–
	Significant	–	–	*p* = 0.016	*p* = 0.006	–	*p* = 05	–	–	–	–	–
	CI_95_		–	−1.75; −0.181	−0.447; −0.076	–	−0.42; −0.001	–	–	–	–	–
Status: single (single, divorced, widower)	Redundant											
Education: Elementary school	B	–	−4.31	–	–	2.163		2.37	1.56	–	–	–
	Wald χ^2^	–	6.324	–	–	10.51		15.19	8.1	–	–	–
	Significant	–	*p* = 0.012	–	–	*p* = 0.001		*p* < 0.001	*p* = 0.004	–	–	–
	CI_95_		−7.76; −0.951	–	–	0.855; 3.47		1.178; 3.56	0.486; 2.63	–	–	–
Education: Middle-school	B	–	–	–	–	0.776	–	–	0.786	–	0.95	0.447
	Wald χ^2^	–	–	–	–	8.79	–	–	13.4	–	9.63	9.52
	Significant	–	–	–	–	*p* = 0.003	–	–	*p* < 0.001	–	*p* = 0.002	*p* = 0.002
	CI_95_		–	–	–	0.263; 1.29	–	–	0.365; 1.207	–	0.333; 1.477	0.163; 0.732
Education: High-school	B	4.59	–	1.672	–	–	–	0.395	0.382	0.243	0.45	0.229
	Wald χ^2^	15.88	–	11.69	–	–	–	10.821	12.93	4.12	9.39	9.604
	Significant	*p* < 0.001	–	*p* = 0.001	–	–	–	*p* = 0.001	*p* < 0.001	*p* = 0.042	*p* = 0.002	*p* = 0.002
	CI_95_	2.31–6.77	–	0.685; 2.7	–	–	–	0.15; 0.63	0.177; 0.600	0.008; 0.47	0.162; 0.738	0.083; 0.369
Education: Bachelor	B	–	–	1.183		0.324	–	–	0.376	–	0.45	0.219
	Wald χ^2^	–	–	4.289		4.71	–	–	9.38	–	6.962	7.013
	Significant	–	–	*p* = 0.046		*p* = 0.03	–	–	*p* = 0.002		0.007	*p* = 0.008
	CI_95_	–	–	0.022; 2.26		0.031; 0.617	–	–	0.135; 0.616		0.113; 0.766	0.057; 0.382
**Predictors**	**Statistics**	**Anxiety**	**PA**	**NA**	**SB**	**KNW**	**VUL^*a*^**	**CON**	**SEV**	**TER**	**DFG**	**COM**
Education: Master	B	*–*	–	–	–	–	–	–	0.273	–	–	–
	Wald χ^2^	*–*	–	–	–	–	–	–	5.76	–	–	–
	Significant	*–*	–	–	–	–	–	–	*p* = 0.016	–	–	–
	CI_95_		–	–	–	–	–	–	0.05; 0.495		–	–
Education: P.hd./MS	Redundant											
Number of children (no children)	B	*–*	−1.287		–	–		–	0.46	–	0.932	–
	Wald χ^2^	*–*	3.6		–	–		–	4.42	–	10.02	–
	Significant	–	*p* = 0.058		–	–		–	*p* = 0.036	–	0.002	–
	CI_95_	–	−2.62; 0.043		–	–		–	0.031; 0.881	–	0.355; 1.51	–
Number of children (one children)	B	–	−1.369		–	–		–	0.45	–	0.896	–
	Wald χ^2^	–	2.784		–	–		–	4.5	–	9.21	–
	Significant		4.05		–	–		–	*p* = 0.033	–	0.002	–
	CI_95_	–	−2.7; −0.035		–	–		–	0.038; 0.89	–	0.317; 1.47	–
Number of children (two children)	B	–			–	–	–	–	–	–	0.794	–
	Wald χ^2^	–			–	–	–	–	–	–	7.418	–
	Significant	–			–	–	–	–	–	–	0.006	–
	CI_95_				–	–	–	–	–	–	0.223; 1.366	–
Number of children (three or more)	Redundant											
Residency: countryside	B		–	–	–	–	–	–	–	–	–	–
	Wald χ^2^		–	–	–	–	–	–	–	–	–	–
	Significant		–	–	–	–	–	–	–	–	–	–
	CI_95_		–	–	–	–	–	–	–	–	–	–
Residency: town	B	–	–	–	–	–	–	–	–	–	–	–
	Wald χ^2^	–	–	–	–	–	–	–	–	–	–	–
	Significant	–	–	–	–	–	–	–	–	–	–	–
	CI_95_		–	–	–	–	–	–	–	–	–	–
Residency: suburbs	B	–	–	–	–	–	–	–	–	–	–	–
	Wald χ^2^	–	–	–	–	–	–	–	–	–	–	–
	Significant	–	–	–	–	–	–	–	–	–	–	–
Residency: city centre	Redundant											
Residency in Italy: Northern Italy	B	–	–	–	–	–	–	–				
	Wald χ^2^	–	–	–	–	–	–	–	–	–	–	–
	Significant	–	–	–	–	–	–	–	–	–	–	–
Residency in Italy: Central Italy	B	–	–	–	–	–	–	–	–	–	–	–
	Wald χ^2^	–	–	–	–	–	–	–	–	–	–	–
	Significant	–	–	–	–	–	–	–	–	–	–	–
**Predictors**	**Statistics**	**Anxiety**	**PA**	**NA**	**SB**	**KNW**	**VUL^*a*^**	**CON**	**SEV**	**TER**	**DFG**	**COM**
Residency in Italy: Southern Italy	B	–	–	–	–	–	–	–	–	–	–	–
	Wald χ^2^	–	–	–	–	–	–	–	–	–	–	–
	Significant	–	–	–	–	–	–	–	–	–	–	–
Residency in Italy: Islands	Redundant											
Age	B	−0.104	0.024	−0.062	−0.020	0.015	–	0.016	0.009	−0.014	–	−0.011
	Wald χ^2^	6.49	3.84	12.02	18.68	10.28	–	13.083	5.22	10.46	–	15.995
	Significant	*p* = 0.011	*p* = 0.05	*p* = 001	*p* < 0.001	*p* = 0.001	–	*p* < 0.001	*p* = 0.022	*p* = 001	–	*p* < 0.001
	CI_95_	−0.186; −0.024	−0.0,3-e; 0.049	−0.99; −0.028	−0.027; −0.01	0.006; 0.025	–	0.007; 0.24	0.001; 0.017	−0.023; −0.006	–	−0.016; −0.005

*Note. “male” as category of “gender”; “single” as a category of “status”; “Ph.d,/MS” as a level of “Education”; “three or more children” as “Number of children”; “city center” as a level of “Residency”; “Islands” as a level of “Residency in Italy” were not reported since they are a redundant levels. Marital status was transformed into a dummy variable (engaged vs. single). ^*a*^The predictor was significant, but the omnibus model was not. CI95, conventional 95% confidence interval; PA, positive affect; NA, negative affect; SB, search behavior; KNW, knowledge; VUL, vulnerability; CON, control; SEV, severity; TER, terrifying; DFG, damage for future generations; COM, compliance.*

We reported results for each of the dependant variables (anxiety, PA, NA, SB, KNW, VUL, CON, SEV, TER, DFG, COM) in relation to all predictors taken together (gender, marital status, education, number of children, residency, residency in Italy). Only B values useful for explaining results were reported in order to avoid redundancies.

Younger females (Age: B = −0.104) with lower education (high school: B = 4.59) are related with highest levels of anxiety. Regarding PA, being male (gender: B = −1.367), with lower number of children (number of children: beta decreased from one to no children but with a negative value: B = −1.369 to −1.287) and older (B = 0.024) significantly increased PA. Indeed, NA was significantly positively predicted by being a woman, not engaged (engaged marital status: B = −0.963) with lower education (from bachelor = 1.183 to high school: B = 1.672) and younger (B = −0.062). Younger (B = −0.26) and single people (gender did not result as a significant predictor) positively predicted the frequency of online health information searching behavior. Being female (B = 12.75), with a lower level of education (B = 2.163–0.776) and senior (B = 0.015), led to significantly higher perception of risk knowledge. Only being single (B = −0.21), i.e., not engaged, significantly positively predicted the perception of being vulnerable against risk. A lower education (beta decreased positively from high school to elementary school) and being older (B = 0.016) significantly positively predicted the perception of control over the risk associated with the pandemic. Being female, with a lower level of education, and with no to one child and older led to a significantly higher perception of risk severity. Being less educated and younger led to a significantly higher perception of risk as terrifying. Being female, with lower education, and an increasing number of children (from no to two children) led to a significantly higher perception of risk damage associated with new generations. Females with lower education and younger tended to report more compliance toward the government’s measures.

### Path Analysis

Descriptive statistics for all the measures and zero-order correlations between them are described in Table 2. With the aim of exploring a measurement model and construct validity, a confirmatory factor analysis (CFA) was conducted comparing four nested models from one factor to a final model composed of the four principal latent factors (risk perception, NA, PA, and anxiety). Table 3 represents Chi-square and goodness of fit indices for the four measurement models developed. Considering that risk perceptions were all measured with single items, and despite the final CFA indexes not being optimal, there was an evident amelioration of all indices from the first to the final model. Therefore, the measurement model can be profitably used in further testing of the proposed structural model.

**TABLE 2:** Descriptive statistics and zero-order correlations among the variables of the study.

	***M* (SD)**	**1**	**2**	**3**	**4**	**5**	**6**	**7**	**8**	**9**	**10**
1. Uncontrollability	3.87 (1.7)										
2. Terrifying	4.89 (1.3)	0.061									
3. Fatality	3.37 (1.2)	0.143**	0.095**								
4. Danger for future generation	3.31 (1.7)	0.125**	0.068*	0.477***							
5. Vulnerability	3.67 (1.5)	0.254***	0.043	0.214**	0.223***						
6. Risk knowledge	13.84 (5.7)	−0.056	−0.009	−0.086*	−0.026	−0.006					
7. Negative affect	11.83 (3.8)	0.164**	0.180**	0.263***	0.308***	0.237***	−0.022				
8. Positive affect	10.46 (2.6)	−0.163**	−0.036	−0.099**	−0.047	−0.086**	0.155**	−0.387***			
9. Anxiety	1.77 (0.83)	0.189**	0.117**	0.227***	0.263***	0.228***	−0.053	0.858***	−0.597***		
10. Complaint	2.4 (1.3)	0.008	0.148**	0.141**	0.124**	0.133**	0.029	0.153**	−0.047	0.166**	
11. Search behavior	3.87 (1.7)	0.068*	0.056	0.001	0.007	0.091**	0.171**	0.264***	−0.103**	0.246***	0.069*

***p < 0.001; **p < 0.01; *p < 0.05.

**TABLE 3:** Goodness of fit indices of the alternative measurement models on measured variables.

	**Chi-square**	**df**	**RMSEA**	**CFI**	**IFI**	**SRMR**
Model 1 – one factor	7205.047	560	0.154	0.734	0.724	0.113
Model 2 – two factors	6488.564	559	0.134	0.792	0.793	0.096
Model 3 – three factors	5720.552	557	0.101	0.853	0.843	0.089
Model 4 – four factors	5438.563	554	0.089	0.904	0.898	0.081

*df, degrees of freedom; RMSEA, root mean square error of approximation; CFI, comparative fit index; IFI, incremental fit index; SRMR, standardized root mean square residual.*

**FIGURE 3:**
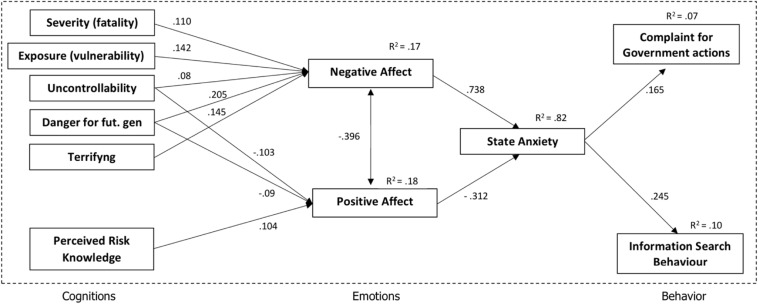
Final structural model on study variables.

Thus, we tested through SEM the proposed structural model (Figure 2): the five risk perception dimensions (fatality, vulnerability, uncontrollability, terrifying) and risk knowledge as (correlated) antecedents, with direct relationships with both NA and PA as intermediate variables, which themselves have direct links with state anxiety that fully mediates information search behavior. The proposed model exhibited optimal goodness of fit: Chi-square = 112.812 (df = 23; *p* < 0.000), RMSEA = 0.063, CFI = 0.966, IFI = 0.967, NFI = 0.958, GFI = 0.980, TLI = 0.919. Consequently, we tested the same model deleting nonsignificant relationships (severity, vulnerability, and terrifying with PA; risk knowledge with NA) and some correlations between antecedents (vulnerability and danger for future generations, with terrifying risk, risk dimensions, and risk knowledge). Consistent with our hypothesized relationships, the model showed excellent goodness of fit: Chi-square = 129,737 (df = 33; *p* < 0.000), RMSEA = 0.054, CFI = 0.964, IFI = 0.964, NFI = 0.952, GFI = 0.977, TLI = 0.94, with all significant relationships (*p* < 0.001). Regression weights are presented in Table 4, while the path diagram of the final model is shown in Figure 3.

**TABLE 4:** Standardized path coefficient (regression weights) of the final model.

**Estimate**			
Negative affect	←	Severity	0.110
Negative affect	←	Vulnerability	0.142
Negative affect	←	Uncontrollability	0.080
Negative affect	←	Danger for future generation	0.205
Negative affect	←	Terrifying	0.145
Positive affect	←	Knowledge	0.104
Positive affect	←	Damage for future generation	−0.090
Positive affect	←	Uncontrollability	−0.103
State anxiety	←	Negative affect	0.738
State anxiety	←	Positive affect	−0.312
Search behavior	←	State anxiety	0.245
Complaint	←	State anxiety	0.165

As hypothesized (Hp2a), each dimension of risk perception is positively related to NA. In contrast, only two dimensions (uncontrollability and danger for future generations) are significantly linked to PA, not confirming what was expected (Hp2b); conversely, confirming Hypotheses Hp3a but not Hp3b, risk knowledge is only positively related to PA. Results confirmed that the expected differential effect of NA (Hp4a) and PA (Hp4b) on search behavior and on a complaint is fully mediated by state anxiety (PA indirect effect on search behavior: β = −0.051, *p* < 0.001; NA indirect effect on search behavior: β = 0.142, *p* < 0.001). Overall, the relationships expressed in the model explained 17% of the variance for NA, 18% for PA, 82% for state anxiety, 10% for search behavior, and 7% for complaint in government actions.

## Discussion and Conclusion

The present research carried forward the literature regarding the fact that cognitive factors predict population affect that, in turn, motivate and fully mediate information search behavior and complaints about government actions, overturning contributions that proposed that risk behavior is driven by affects (Kahan, 2008; Leppin and Aro, 2009; Wu et al., 2018).

Considering our sample of participants, results showed that being female and younger with a lower level of education led to more anxiety, NA, a higher risk perception as terrifying, and higher complaint regarding GR. Moreover, PA increased significantly in older males and those with a decreasing number of children (less than two). Younger people or those who were not engaged or married tended to look for information online about the COVID-19 more frequently. Older females with a lower education level (from middle to elementary school) were more prone to perceive themselves as competent regarding their acquired knowledge on COVID-19. Being engaged or married acted as a protective factor regarding the perceived vulnerability against COVID-19. Older people with lower levels of education (from high school to elementary school degree) tended to feel more able to control the gravity of risks associated with this pandemic. Older females having from one to no children, and with high-school to elementary school degree, tended to perceive the COVID-19-associated risk as more severe. Females who have a higher number of children (from no child to two children) and with a lower level of education (this effect increased from bachelor to elementary school) tented to perceive an increased risk associated with COVID-19 for future generations. Younger females with lower education tended to report more compliance toward the government’s measures.

### Theoretical Implications

In this study, we elucidated two crucial phenomena in emergency: general NA and its link with anxiety. NA and anxiety have often resulted in closely positively intertwined affective states (Crawford and Henry, 2004) even though they can be considered as clear, distinguishable constructs (Watson and Kendall, 1989; Clark and Watson, 1991). The Tripartite model of anxiety and depression confirmed that high levels of NA underlie both anxiety and depression, while NA acts as a predictor of anxiety (Clark and Watson, 1991). Specifically, NA has been often considered as an early predictor of anxiety in several domains (Crawford and Henry, 2004; Cisler et al., 2010). The model tested in this study confirmed the direction of this link.

Emotions and affect also act as motivators of specific survival behaviors (Frijda et al., 1989), and this definition could hardly be more appropriate than in this worldwide emergency. In this case, affect can trigger behaviors such as online health information seeking or avoidance (Savolainen, 2014). While PA resulted in playing a pivotal role in determining people’s attitudes toward information avoidance, the negative one predicted individuals’ attitudes toward information seeking (Yang and Kahlor, 2013). On the other hand, searching for information about symptoms or specific illnesses can increase people distress and anxiety about their health following a reinforcing spiral to the extent that a new term has been coined to refer to this condition, i.e., “cyberchondria” (Te Poel et al., 2016). Indeed, people with high health anxiety (i.e., fears stemming when individuals exaggerate in interpreting their bodily symptoms as an indicating severe illnesses) (McMullan et al., 2019) trend to increase their negative responses related to the likelihood of suffering from a given disease now and in the future (Baumgartner and Hartmann, 2011).

The present result showed that anxiety triggered by NA acted as a strong predictor of people’s searching behavior regarding health. In other words, Italians were motivated by anxiety stemming from NA and triggered by their risk perception on the controllability and vulnerability regarding SARS-CoV-2 spread and health searching behavior. Overall, results provided support for the cognitive appraisal framework in risk perception (Roseman et al., 1996; Keller et al., 2012) and the main hypotheses. Risk perception and knowledge acted with different mechanisms on emotions: risk perception mainly contributed to having an effect on negative affect, while knowledge influenced only positive affect. Furthermore, in line with our hypotheses, emotions fully mediated the relation among risk cognition, complaint, and information search behavior (Champion and Skinner, 2008; Carpenter, 2010; Jung et al., 2015).

### Policy Implications

Risk perception and affective response to pandemics can be crucial factors for managing population behaviors, thus ensuring the best adherence to prescription and safety norms (Poletti et al., 2011; Merino, 2014; Shook et al., 2019). Moreover, the efficiency of prevention behaviors in pandemics by the Government is related to population cooperation, which is highly related to risk perception (Leppin and Aro, 2009; Goodwin et al., 2011). Exploring risk perception during pandemics is fundamental because misperceptions can often cause inadequate responses (Poletti et al., 2011; Merino, 2014). In particular, perceptions regarding infection can lead people to take safer actions, to reduce exposure, and to increase protective conducts (e.g., vaccination, social distancing, hygiene, search for information; Shook et al., 2019). These individual behaviors can significantly influence the disease progression at a system level (De Zwart et al., 2007; Jiang et al., 2009).

Since emotion and behavior are closely related (Loewenstein et al., 2001; Slovic and Peters, 2006; Brown, 2014), beliefs and perceptions regarding risk represent core predisposing factors to predict people reactions. Therefore, it would be crucial to promote public order and right risk communication and to prevent counterproductive behaviors linked to bad information and fake news (Brug et al., 2004; Voeten et al., 2009; Shook et al., 2019).

The risk controllability is one of the most important factors that need to be considered since PA can reduce anxiety and, consequently, affect complaint and informational search behavior. In Italy, especially during the first days of the epidemic (from the end of February), there was too much conflicting information (e.g., “This virus is very similar to normal flu.”/“Please, pay attention, it is a very dangerous virus; it is not like normal flu.”).

It is crucial to evidence that too much information, especially if conflicting (or worse, fake news), can cause confusion in the population, and this, in turn, can affect emotional states (Bawden and Robinson, 2009). Politicians should act on proper information dispersion procedures regarding specific risk, as perceived knowledge may act on search behavior and complaint. Our results can suggest more tailored strategies of communication for prevention to be implemented by the government, not just in pandemic emergency (Smith, 2006).

Research regarding the way the population appraised hazards acquired significant scientific attention, and different approaches and paradigms to the perception of risk have been discussed (Leppin and Aro, 2009; Keller et al., 2012). Recent contributions have conformed on the emotional appraisal of risk perception (Loewenstein et al., 2001; Wu et al., 2018). Thus, results from our study could offer evidence in favor of the hypothesis that the analytic system (i.e., risk judgment) would precede the emotional one, at least in a pandemic emergency.

### Country-Level Implications

COVID-19 is having, and is predicted, to have a substantial impact on the world economy, both due to the effect on national health systems, and on the slowdown of business activities through lockdowns and measures of social distancing. The economic impact would be even more substantial in developing countries, due to both difficulties related to social distancing in the slums and in the suburbs, as well as for the absence of stable health systems, welfare measures, and smart-working policies, and for the access to the various forms of institutional communication and to the mass media. The literature concerning the other pandemics has clearly shown that the perception of risk has a strong cultural component; thus, communication strategies should be tailored according to the peculiarities of each country (Jiang et al., 2009). In this regard, the proposed model can indicate a priority of all the variables capable of influencing preventive behavior or adherence to restrictions directly, which must be taken into account when planning communication to the general public. For instance, accurate and clear communication should clarify the danger for future generations, the terror aroused, and the degree of exposure to the pandemic (Van Bortel et al., 2016). Furthermore, the proposed model evidenced also perceived knowledge of risk as another key variable to be considered in mass communication. Finally, communication in developing countries should consider that people living in precarious economic conditions could give less weight to the health consequences of COVID-19, in a cost-benefit assessment process that could overestimate economic costs to the detriment of those for health and economics (Leppin and Aro, 2009).

### Limitations of the Study

Given the novelty and relevance of this study, some limitations should be discussed. First of all, the cross-sectional design of the research limits the generalizability of its findings.

Although results should be interpreted, especially concerning the specificity of both the contagion risk and the quarantine situation, useful indications on the mechanism that operates between cognitions, emotions, and behaviors in situations of high stress and forced captivity can be provided. Moreover, due to the recruitment type (i.e., online), and despite a large number of participants, this sample cannot be considered as fully representative of the Italian population (26% males, 57% in Northern Italy). Almost 50% of the participants filled out the survey in the first 2 days (maybe caused by people’s reactions to the lockdown). Therefore, this distribution does not allow for longitudinal analysis.

## Data Availability Statement

The raw data supporting the conclusions of this article will be made available by the authors, without undue reservation.

## Ethics Statement

Ethical review and approval was not required for the study on human participants in accordance with the local legislation and institutional requirements. The patients/participants provided their written informed consent to participate in this study.

## Author Contributions

AD and MB conceived and designed the experiments. MB and AC done through questionnaires to target groups investigation. MB, AC, and GK analyzed the data. AD contributed the analysis tools. MB, AC, and AD wrote the manuscript. AD and GK critically reviewed the manuscript. All authors contributed to the article and approved the submitted version.

## Conflict of Interest

The authors declare that the research was conducted in the absence of any commercial or financial relationships that could be construed as a potential conflict of interest.
